# Lightning Strike-Induced Cardiac Arrest Managed With Extracorporeal Cardiopulmonary Resuscitation: A Case Report

**DOI:** 10.7759/cureus.92035

**Published:** 2025-09-11

**Authors:** Akira Suekane, Kaori Miyazaki, Natsuko Kawasoe, Shunya Shiraishi, Tatsunori Ameda

**Affiliations:** 1 Department of Emergency Medicine, Miyazaki Prefectural Miyazaki Hospital, Miyazaki, JPN; 2 Department of Acute Critical Care and Disaster Medicine, Institute of Science Tokyo, Tokyo, JPN; 3 Department of Emergency and Critical Care Medicine, University of Miyazaki Hospital, Miyazaki, JPN; 4 Department of General Internal Medicine, Miyazaki Prefectural Miyazaki Hospital, Miyazaki, JPN

**Keywords:** cardiac arrest, ecmo, extracorporeal cardiopulmonary resuscitation, lightning injury, pericardial tamponade

## Abstract

Lightning strike injuries have a high mortality rate and frequently result in immediate cardiac arrest. Although extracorporeal cardiopulmonary resuscitation (ECPR) is increasingly used for out-of-hospital cardiac arrest, its role in lightning strike-induced cardiac arrest remains unclear.

We report the case of a 17-year-old male who experienced cardiac arrest after a direct lightning strike. The initial rhythm was non-shockable, and veno-arterial extracorporeal membrane oxygenation (VA-ECMO) was initiated. Return of spontaneous circulation (ROSC) was achieved 81 minutes after the lightning strike. The patient required extensive fluid resuscitation, including blood products, and subsequently developed a pericardial tamponade that required drainage. Despite complications such as compartment syndrome and gastrointestinal bleeding, the patient survived.

This case highlights the potential utility of ECPR in lightning-induced cardiac arrest and the importance of early recognition of pericardial tamponade. Similar to burn management, aggressive fluid resuscitation is critical. Further research is warranted to optimize the treatment protocols for lightning strike victims.

## Introduction

Lightning injuries are associated with a mortality rate of 10%-30%, with global estimates suggesting 0.2-1.7 deaths per 1,000,000 people annually, varying by region [1]. A significant proportion of casualties are sustained by young people participating in outdoor activities [2]. In Japan, there are reports of one death per year [3]. Although immediate cardiac arrest is common, half of the initial rhythms identified are ventricular fibrillation (VF), and 40% are asystole [4]. Cardiovascular injuries caused by lightning strikes have been reported, including myocardial contusions, Takotsubo cardiomyopathy, and myocardial infarction caused by vasospasm [5]. Myocardial contusions from a lightning strike may present with <15% cardiac function but may be reversible [6]. The application of extracorporeal cardiopulmonary resuscitation (ECPR) has increased worldwide; however, its use in lightning-related injuries has not been well documented, and most applications have been for out-of-hospital cardiac arrest due to internal causes. However, its application in lightning-related cardiac arrest remains extremely rare and has not been well documented. We report the case of a 17-year-old male who experienced cardiac arrest following a direct lightning strike. Despite the initial unshockable rhythm, the patient was successfully resuscitated using veno-arterial extracorporeal membrane oxygenation (VA-ECMO) but required substantial fluid resuscitation.

## Case presentation

**Figure 1:**
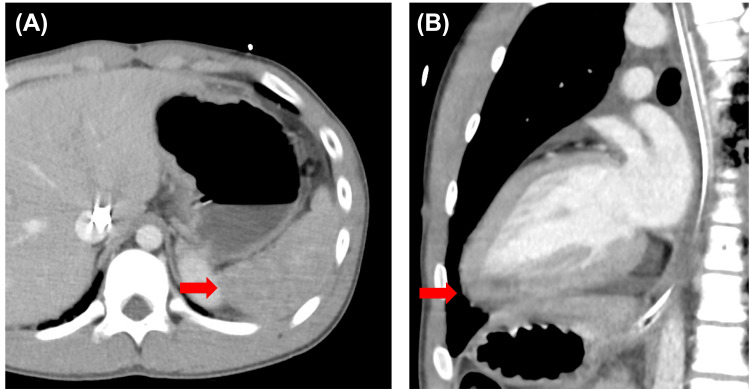
Contrast-enhanced computed tomography showing splenic ischemia and myocardial injury after a lightning strike Contrast-enhanced CT images demonstrating (A) splenic ischemia (arrow) and (B) poor contrast enhancement in the lateral and inferior walls of the left ventricle (arrow), suggesting myocardial injury likely caused by the lightning strike.

**Figure 2:**
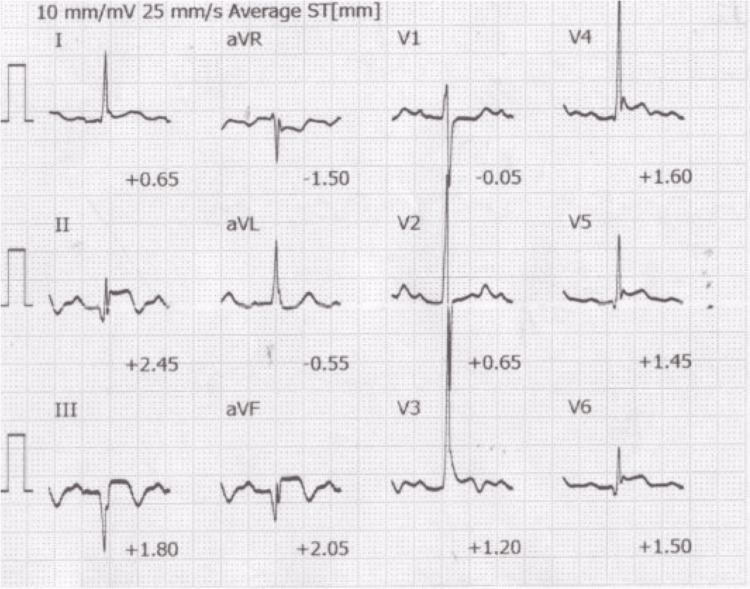
Electrocardiogram on ICU admission Electrocardiogram obtained on ICU admission, showing sinus rhythm with diffuse ST-segment elevations.

A 17-year-old male was admitted to a tertiary emergency and critical care center in a regional city with cardiac arrest following a direct lightning strike. Bystander cardiopulmonary resuscitation was initiated immediately after the collapse, which was considered the onset of low-flow circulation. The no-flow time was approximately seven minutes, and the total low-flow time until VA-ECMO initiation was estimated to be 52 minutes. The estimated time from onset to hospital arrival was 40 minutes. The subsequent resuscitation sequence was as follows: physician contact by the doctor car team occurred 18 minutes after the strike with asystole, intubation was performed at 21 minutes, VF appeared at 34 minutes, and was treated with defibrillation, followed by pulseless electrical activity. Ventricular tachycardia (VT) was observed at 40 minutes and defibrillated. The patient arrived at the catheterization laboratory at 43 minutes with persistent VT. At 55 minutes, 300 mg of amiodarone and a cumulative total of 13 times of 1 mg epinephrine had been administered. VA-ECMO was initiated at 59 minutes after the strike. At 81 minutes after the strike, spontaneous circulation returned while the ECMO pump was running. After return of spontaneous circulation (ROSC), the patient remained hypotensive and required vasoactive support with norepinephrine. Targeted temperature management was initiated and maintained for 48 hours. Continuous hemodynamic monitoring was performed in the ICU, and additional organ support, including intra-aortic balloon pumping and renal replacement therapy, was provided as needed. Computed tomography scans showed splenic ischemia (Figure 1A) along with areas of poor contrast enhancement in the lateral and inferior walls of the left ventricle (Figure 1B), suggesting myocardial injury likely caused by the lightning strike. An ECG obtained on ICU admission demonstrated sinus rhythm with diffuse ST-segment elevations (Figure 2).

The patient exhibited severe mixed acidosis and elevated lactate levels (Table 1).

**Table 1:** Initial laboratory findings on admission Initial laboratory values were obtained upon admission following a lightning strike. WBC: white blood cell count; RBC: red blood cell count; Hb: hemoglobin; HCT: hematocrit; MCV: mean corpuscular volume; MCHC: mean corpuscular hemoglobin concentration; Plt: platelet count; Neut: neutrophils; Lym: lymphocytes; PT-INR: prothrombin time-international normalized ratio; APTT: activated partial thromboplastin time; T-Bil: total bilirubin; AST: aspartate aminotransferase; ALT: alanine aminotransferase; LD: lactate dehydrogenase; ALP: alkaline phosphatase; γGTP: gamma-glutamyl transpeptidase; S-Amy: serum amylase; CK: creatine kinase; CK-MB: creatine kinase-myocardial band; Troponin I: cardiac troponin I; BUN: blood urea nitrogen; CRP: C-reactive protein; pH: acidity/alkalinity of blood; pO₂: partial pressure of oxygen; pCO₂: partial pressure of carbon dioxide; HCO₃: bicarbonate

Blood Test	Patient Value	Reference Range
WBC (×10^3^/μl)	6.78	3.3–8.6
MCHC (%)	32.8	31.7–35.3
MCV (%)	92.6	83.6–98.2
HCT (%)	43.6	40.7–50.1
Hb (g/dl)	14.3	13.7–16.8
RBC (×10^6^/μl)	4.71	4.35–5.55
Plt (×10^3^/μl)	239	158–348
Neut (%)	28.8	38–74
Lym (%)	68.9	16.5–49.5
PT-INR	1.81	0.90–1.15
APTT	>360	24–39
Fibrinogen (mg/dl)	133.7	200–400
D-dimer (μg/ml)	29.3	0–1
T-Bil (mg/dl)	0.95	0.4–1.5
AST (U/L)	331	13–30
ALT (U/L)	203	10–42
LD (U/L)	927	124–222
ALP (U/L)	117	38–113
γGTP (U/L)	16	13–64
S-Amy (U/L)	992	44–132
CK (U/L)	3641	59–248
Glucose (mg/dl)	87	73–109
Sodium (mmol/L)	146	138–145
Potassium (mmol/L)	4	3.6–4.8
Calcium (mg/dl)	9.6	8.8–10.1
Chloride (mmol/L)	99	101–108
BUN (mg/dl)	24.5	8–20
Creatinine (mg/dl)	1.45	0.65–1.07
CRP (mg/dl)	0	0–0.14
Troponin I (ng/ml)	>50	0–0.026
CK-MB (U/L)	589	0–12
Blood Gas		
pH	7.185	7.35–7.45
pO2 (mmHg)	469	75–100
pCO2 (mmHg)	35	35–45
HCO3 (mEq/L)	13.2	20–26
Lactate (mmol/L)	23	0.5–2

**Figure 3:**
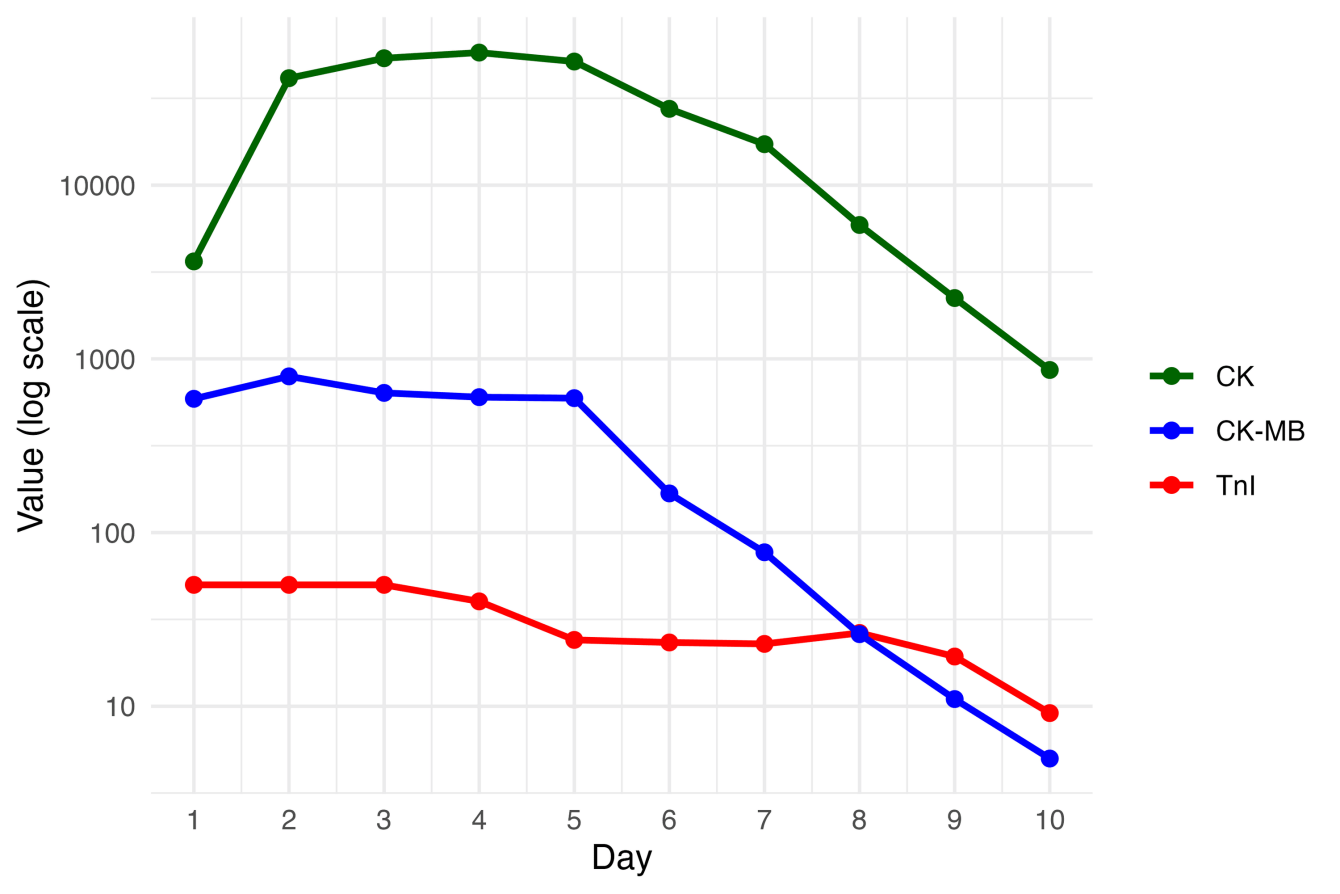
Serum CK, CK-MB, and troponin I levels after the lightning injury Serum levels of creatine kinase (CK), creatine kinase–myocardial band (CK-MB), and troponin I (TnI) following lightning injury. Values are plotted on a logarithmic scale. CK is shown in dark green, CK-MB in blue, and TnI in red.

Blood tests revealed markedly elevated cardiac and muscle biomarkers on admission, including troponin I (>50 ng/ml), creatine kinase-myocardial band (CK-MB) (589 U/L), and CK (3641 U/L). The peak CK levels were observed on day 4, while troponin I gradually decreased over the first week (Figure 3).

Even after ECMO insertion, the ejection fraction remained at 10%-20%. An intra-aortic balloon pump was inserted due to decreased myocardial contractility; norepinephrine was administered at 0.3 γ, and continuous renal replacement therapy was initiated for severe acidemia.

**Figure 4:**
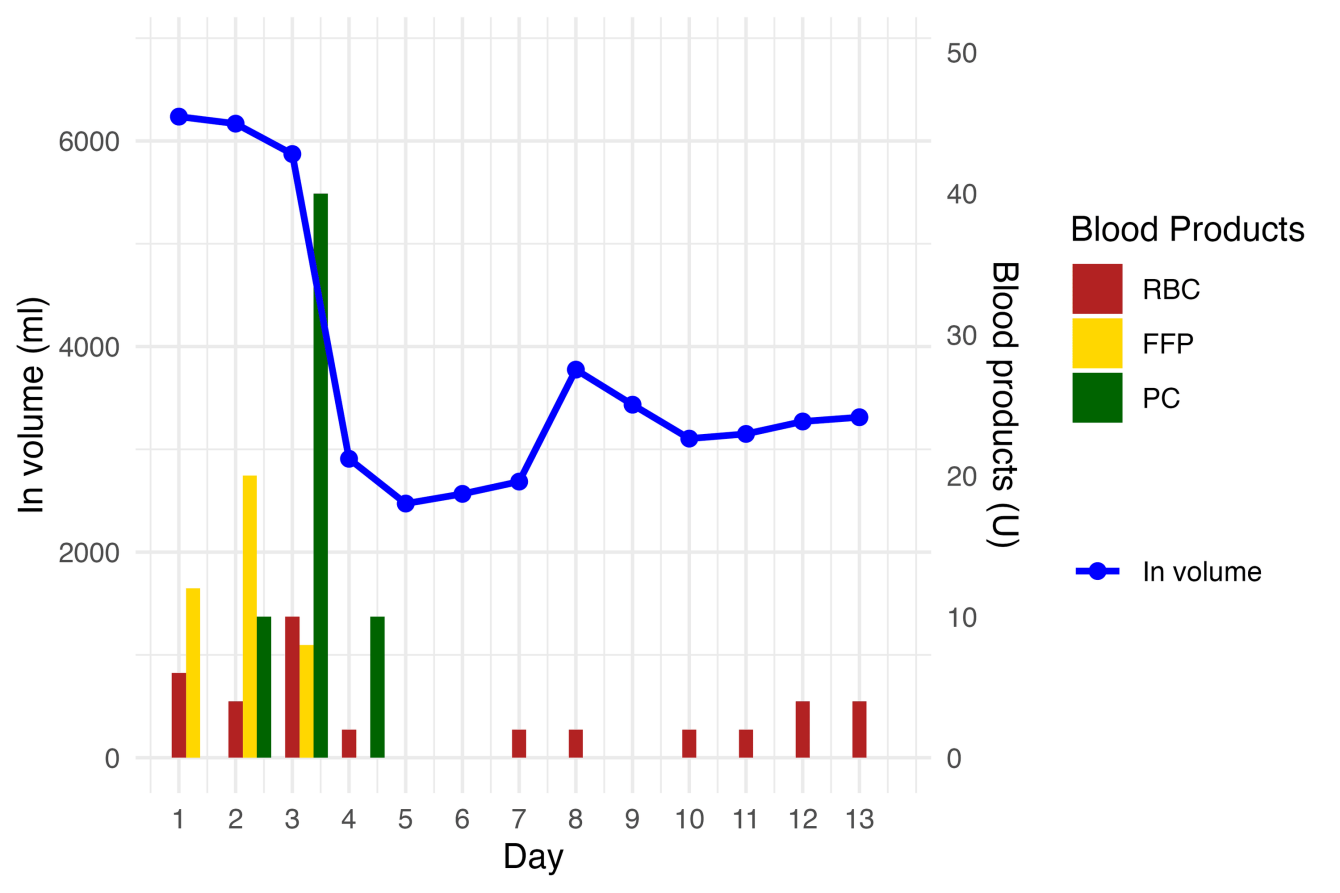
Daily fluid balance and transfusion volume (dual axis) Daily fluid input volume and transfused blood product units. Fluid input is shown as a blue line on the left y-axis (ml). Transfused red blood cells (RBC), fresh frozen plasma (FFP), and platelet concentrates (PC) are shown as red, gold, and dark green bars, respectively, scaled to the right y-axis (units). The maximum transfusion axis is capped at 50 units.

The first 24 hours were critical; the patient required a total volume of approximately six liters per day for the first three days, including fluids and blood products such as crystalloids, fresh frozen plasma, and red blood cells (Figure 4).

**Figure 5:**
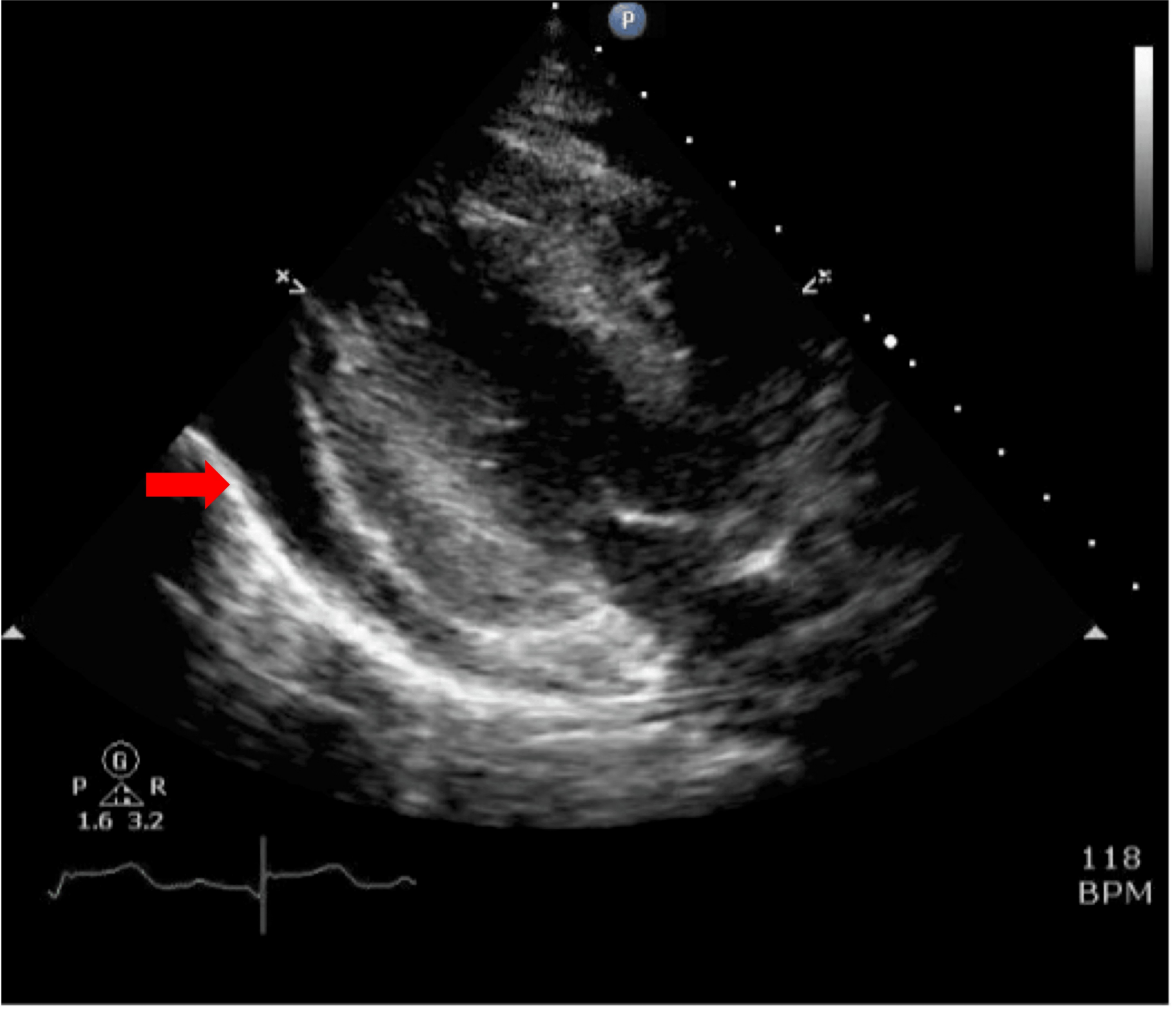
Echocardiography on ICU day 2 Transthoracic echocardiography was performed on ICU day 2, several hours before pericardial drainage. The arrow indicates pericardial effusion.

On the second day, pericardial effusion leading to tamponade required drainage (Figure 5).

The fluid was serous and likely exudative from the cardiac thermal injury, with laboratory analysis showing specific gravity 1.028, protein 3.2 g/dL, LDH 4397 U/L, glucose 100 mg/dL, and lymphocyte predominance. Following drainage, cardiac function improved, vascular permeability stabilized, and the need for infusion significantly decreased. ECMO was discontinued on day 3. Despite complications such as muscle necrosis and compartment syndrome in the lower limbs that required fasciotomy and partial debridement, the patient survived. Subsequent gastrointestinal bleeding was managed successfully with interventional radiology, and the patient was transferred to another hospital on day 23 and subsequently to a rehabilitation hospital on day 68. At the time of rehabilitation transfer, his cerebral performance category (CPC) remained four.

## Discussion

This case highlights the potential utility of ECPR for lightning-induced cardiac arrest, even when the initial rhythm is unshockable. Early recognition and intervention for complications, such as pericardial tamponade, combined with aggressive fluid resuscitation, were key factors in patient survival in this case, despite severe multisystem injuries. While early assumptions pointed to asystole as the predominant rhythm following lightning injury, more recent evidence suggests that ventricular arrhythmias, including VT and VF, are actually more prevalent [7]. The cardiovascular effects of lightning injuries include myocardial contusion, Takotsubo cardiomyopathy, and myocardial infarction [5]. Temporary myocardial contusions can improve, and ECPR may be a reasonable intervention in cases of VF or circulatory failure due to contusions. Conventional resuscitation may be insufficient to restore stable circulation in such settings. ECPR provides immediate circulatory support and maintains systemic perfusion, thereby allowing time for myocardial recovery. Serial measurements of troponin I and CK-MB reflected severe myocardial injury, while the marked elevation and delayed peak of CK indicated extensive skeletal muscle damage due to electrical injury. These biomarker trends provided valuable insights into the temporal progression of tissue injury and recovery. The asystole observed in our patient might have been attributable to prolonged hypoxia, rather than being a direct result of the lightning strike.

ECPR facilitates myocardial recovery and stabilization. Pericardial tamponade developed within 24 hours but resolved quickly, suggesting that peak organ edema might have occurred within this period. This case provides valuable insights into the temporal dynamics of the injury response to lightning strikes and supports the need for further case studies to refine treatment protocols.

The patient in this case had a CPC score of four; each additional minute of no-flow time was associated with a 13% decrease in favorable neurological outcomes [8]. However, reports indicate that young patients with a low flow time (<60 minutes) have a 25% chance of good neurological recovery [9]. Although natural disasters pose challenges to emergency response, some reports have indicated improved consciousness despite prolonged no-flow times, suggesting that ECPR may be justified in certain cases [1,10].

This case highlights the occurrence of pericardial effusion and tamponade in severe lightning injuries, suggesting that physicians should consider this possibility in addition to myocardial contusion. The patient required massive fluid resuscitation within the first 24 hours. Burns typically require the highest fluid volume within 24 hours, after which the requirements decrease [11]. Lightning injuries are managed using Parkland's formula; however, the efficacy of this approach remains unclear [12]. Although the estimated surface burn area was 2%, which does not typically require resuscitative fluids, extensive internal burns from electrical injuries may have led to an underestimation of the fluid requirements.

## Conclusions

Lightning-strike injuries require a multidisciplinary approach for effective management. This case demonstrates the successful use of ECPR and fluid resuscitation strategies adapted from burn care to treat a lightning-strike injury, emphasizing the need for aggressive and tailored interventions in rare but critical situations. Further research and documentation of similar cases are essential to enhance outcomes and develop optimized care protocols for lightning strike victims.
